# Elucidating the Effects of Reaction Time on the Physicochemical Characterization of Valorized Synthesized Alumina

**DOI:** 10.3390/ma15093046

**Published:** 2022-04-22

**Authors:** Aiman A. Bin Mokaizh, Jun Haslinda Shariffuddin, Abdullah O. Baarimah, Amin Al-Fakih, Abdullah Mohamed, Salem O. Baarimah, Al-Baraa Abdulrahman Al-Mekhlafi, Hamoud Alenezi, Olusegun Abayomi Olalere, Anwar Ameen Hezam Saeed

**Affiliations:** 1 Faculty of Chemical and Process Engineering Technology, Universiti Malaysia Pahang, Gambang 26300, Pahang, Malaysia; junhaslinda@ump.edu.my; 2 Department of Civil and Environmental Engineering, Universiti Teknologi PETRONAS, Seri Iskandar 32610, Perak, Malaysia; abdullah_20000260@utp.edu.my; 3 Interdisciplinary Research Center for Construction and Building Materials, King Fahd University of Petroleum and Minerals, Dhahran 31261, Saudi Arabia; aminali.fakih@kfupm.edu.sa; 4 Research Centre, Future University in Egypt, New Cairo 11835, Egypt; mohamed.a@fue.edu.eg; 5 Department of Petroleum Engineering, College of Engineering and Petroleum, Hadhramout University, Al Mukalla 50512, Hadhramout, Yemen; s.baarimah@hu.edu.ye; 6 Department of Management & Humanities, Universiti Teknologi PETRONAS, Seri Iskandar 32610, Perak, Malaysia; albaraa901@gmail.com; 7 Research Institute for Sustainable Environment, School of Chemical and Energy Engineering, Universiti Teknologi Malaysia, Skudai 81310, Johor, Malaysia; homoudkt@gmail.com; 8 ABrC-Inkubator Inovasi Universiti (I2U), sains@usm, Universiti Sains Malaysia, Bayan Lepas 11900, Penang, Malaysia; olabayor@gmail.com; 9 Department of Chemical Engineering, Universiti Teknologi PETRONAS, Seri Iskandar 32610, Perak, Malaysia; anwar_17006829@utp.edu.my

**Keywords:** aluminum oxide, aluminum waste can, characterization, sol–gel synthesis

## Abstract

Aluminum waste-can management in Malaysia has recently become a serious environmental and public health issue, particularly in metropolitan areas. This has prompted the need to valorize these waste-cans into value-added products using the most economical and environmentally friendly techniques. In this study, the sol–gel technique was used to synthesize high-quality alumina from the aluminum waste-cans collected. From this method, the observed peaks of the synthesized alumina were identified as diaspore (α-AlO(OH)), boehmite (γ-AlO(OH)), aluminum oxide, or gamma-alumina (γ-Al_2_O_3_) crystalline structure and corundum. The morphological configuration, microstructure, and functional group properties of the synthesized alumina were evaluated. All the synthesized alumina exhibited a non-spherical shape and appeared to have hexagonal-like shape particles. Moreover, the XRD patterns of the synthesized alumina AL-6-30 and AL-12-30 exhibited a small angle (1–10°) with no XRD peak, which indicated a mesoporous pore structure with no long-range order. The overall results of γ-alumina synthesized from the aluminum waste-cans showed an optimal condition in producing a highly structured γ-alumina with excellent surface-area characteristics. The synthesized alumina exhibited stronger and highly crystalline functional characteristics almost comparable with the commercially available brands on the market.

## 1. Introduction

In recent times, the management of waste in Malaysia has become a major environmental and public health problem mostly in urban areas [1]. In 2016 alone, Malaysia generated over 25,000 metric tonnes of household waste per day and a total of 7 million tonnes annually with yearly growth of 3% [2]. Over the last few decades, extensive technological innovations were focused on discovering economically viable and environmentally friendly methods for vaporization and utilization [3,4,5,6]. One of the most economical and environmentally friendly ways of handling some of the hazardous waste is by recycling [7]. However, according to statistics obtained from the Department of Environment Malaysia (DEM), only 2% of municipal solid waste is being recycled with about 42% of it usually incinerated or chemically treated, whereas the rest is dumped into different landfills [2]. The growing demands and flexibility of disposal of waste aluminum, for example, make it the most reusable packaging material, which is a significant addition to its numerous economic and environmental benefits [8,9]. Aluminum waste has the capacity to retain its physicochemical properties throughout the recycling process [3]. The recycling process, therefore, saves precious natural resources, energy, time and money [10]. Adans et al. [11] estimated that recycling saves up to 95% of the overall metal resources; therefore, only 5% of the total greenhouse gas emissions are recorded. Additionally, one of the benefits of using aluminum waste-cans is their ability to transform directly into alumina as by-products. Alumina is essentially useful as a catalytic and industrial absorbent [12]. Owing to its mechanical and chemical properties, alumina demonstrates major industrial uses which have a broad spectrum of uses in nano-electronics and high-temperature functions [13]. This incredible property is mostly attributable to many benefits, including its resilient characteristic acids and bases, which make it resistant to wear and high temperature [12]. For instance, in many industrial uses such as electronics, metallurgy, optoelectronics and ceramic composites, nano-sized alumina, such as α-alumina, are widely used [14,15].

It is important to know that the conventional method of producing alumina is regrettably expensive and harmful to the environment, as it generates bauxite residue or red mud, which consists of insoluble particles [3]. The sol–gel method of synthesizing alumina from the aluminum waste-can is an easy, safe, and rapid production process that saves a lot of energy and time [16,17]. It should be noted that reaction time plays an important role in the physical and chemical properties of the alumina produced [18,19]. Therefore, in this study, we are examining the effects of reaction time on the physicochemical characteristics in the synthesis of alumina. The physicochemical properties of the synthesized alumina were qualitatively analyzed via Fourier-transform infrared spectroscopy (FTIR), X-ray diffraction (XRD), and scanning electron microscopy (SEM).

## 2. Experimental

### 2.1. Materials and Methods

A disposal site in Sungai Ikan, Terengganu, Malaysia, was established for waste aluminum. Sigma-Aldrich supplied the chemical substances potassium hydroxide (KOH) (No 85%) and sulphuric (H_2_SO_4_) (No 98%). The deionized water was obtained from the analytical laboratory of Universiti Malaysia Pahang for solvent dilution and stock solution preparation.

### 2.2. Sol–Gel Synthesis

The γ-alumina (AL-6-30, AL-12-30, and AL-24-30) were synthesized based on the methods used by Bin Mokaizh et al. [18], Adans et al. [11], and Liu et al. [20], with slight modifications. The waste-cans were pulverized and dissolved in potassium hydroxide (3-Molar). The resulting mixture was thereafter filtered, the pH adjusted to 9 using sulfuric acid (1-Molar) and the solution stirred until it became gel-like. The reaction temperature was fixed at 30 °C, whereas the aging time was varied at 6, 12, and 24 h. The aluminum hydroxide gel was separated by centrifugation and washed several times with deionized water., The aluminum hydroxide gel was dried in the oven at 80 °C for 12 h. The dry substance was calcinated into porous alumina in the air at 500 °C for 2 h. The synthesized alumina is denoted as AL-x-y, where x and y represent reaction time and reaction temperature, respectively. The AL-commercial alumina powder was obtained from Acros Organics and compared with the synthesized alumina.

### 2.3. Physicochemical Characterization

In this analysis, the synthesized γ-alumina was briefly analyzed for functional group properties, morphological shifts, and microstructural elucidation. In particular, the TM3030Plus-Model was employed to elucidate both commercial and synthesized aluminum structures (AL-6-30, AL-12-30, and AL-24-30) from the Benchtop Scanning Electron Microscope. In addition, the functional group and crystalline orientation of the synthesized alumina powders were obtained by Fourier Transform Infrared Spectroscopy (FTIR) and X-ray Diffraction (XRD) trials.

## 3. Results and Discussion

### 3.1. Analysis of Functional Group Characteristics of the Synthesized Alumina

**Figure 1:**
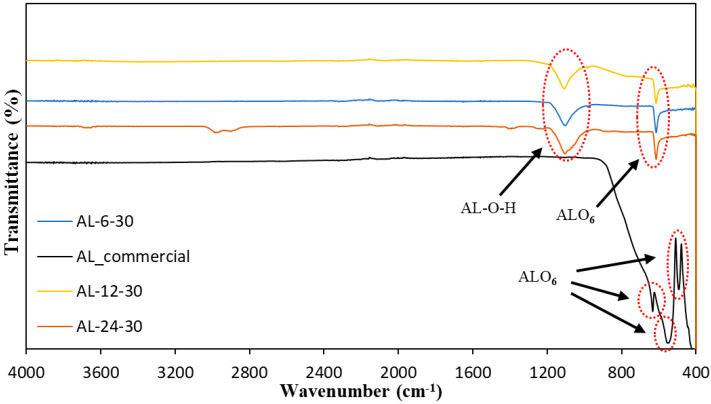
FTIR spectra comparing the effect of reaction times on synthesis of Alumina; AL-6-30, AL-12-30, AL-24-30 and AL-Commercial.

The FTIR spectra from AL-6-30, AL-12-30, AL-24-30, and AL-Commercial are shown in Figure 1. The interpretations of the alumina by the FTIR in the range of 4000 cm^−1^ were carried out, as seen in Figure 1 below. Almost similar FTIR spectrum bands were shared with synthesized alumina. The image of peaks shows that a better-crystallized phase exists.

The ranges were displayed approximately between 629 cm^−1^ and 1100 cm^−1^, whereas the ranges were approximately between 613 cm^−1^ and 1110 cm^−1^ for the alumina AL-12-30 and AL-24-30. For synthesized specimens, AL-6-30, AL-12-30, AL-24-30, the v-AlO_6_ stretch mode was shown at the peak around the band 614 cm^−1^ and 629 cm^−1^. The band was designated to demonstrate the symmetrical deformation of vibrations of Al-O-Al for the synthesized sample around 1100 cm^−1^ and 1110 cm^−1^. Pieces of between 500 and 750 cm^−1^ are assigned to Al-O stretching mode v-AlO_6_, whereas the synthesized samples are designated to v-AlO_4_ deletion mode on the significant shoulder of the 977 to 980 cm^−1^ round [21]. There is no peak of approximately 3500 cm^−1^ and 1600 cm^−1^ that reveals that both samples of γ-Al_2_O_3_ are completely dried of water. Rajaeiyan and Bagheri-Mohagheghi [22] support this by assigning O–H stretching and bending modes of adsorbed water because of the γ-Al_2_O_3_ tendency to spring up with high-intensity bands centered around 3500 cm^−1^ and 1600 cm^−1^. The spectrum shows that Al-O-H has a sharper intensity at peak 614 cm^−1^ with all synthesized alumina AL-6-30, AL-12-30, and AL-24-30. The spectrum of AL-24-30 shows a peak at around 3000 cm^−1^ which indicated Al-OH symmetric bending, which could be due to the synthesized samples exhibiting two different phases. Figure 1 depicted the FTIR spectra bands of AL commercial, which comprise only one homogenous phase in the aluminum hydroxide, at their peaks around 492 cm^−1^, 544 cm^−1^, and 633 cm^−1^ and which represented Al-O stretching mode [23].

The synthesized samples shared similar peaks with AL-commercials which have bands at around 493 to 634 cm^−1^, implying the presence of the Al–O stretching mode of AlO_6_. However, it is the obvious difference at bands around 110 cm^−1^ that related to the Al-O-H symmetric bending which might be due to the synthesized samples having two different phases which are AL-O-H, whereas AL-commercial had one homogenous phase [23]. The spectrum of γ-alumina presented a wide pattern with no apparent thin peak extending from 613 to 980 cm^−1^. The wide range shows that there are amorphous structures or disordered defects. This pattern is characteristic of an intricate crystallography structure assigned as α-Al_2_O_3_ [24]. The vibration of deformation of Al–O–H modes is attributed to the bands from 1100 to 1110 cm^−1^ [25]. Therefore, all synthesized γ-alumina FTIR analysis reveals the formation of a better crystalline phase to the greater intensity of aluminum hydroxide and aluminum oxide. Compared with AL-6-30 and AL-12-30, AL-24-30 had sharper and lengthier ribbons of about 614 cm^−1^ and 1100 cm^−1^.

### 3.2. X-ray Diffraction Patterns of the Synthesized Alumina

**Figure 2:**
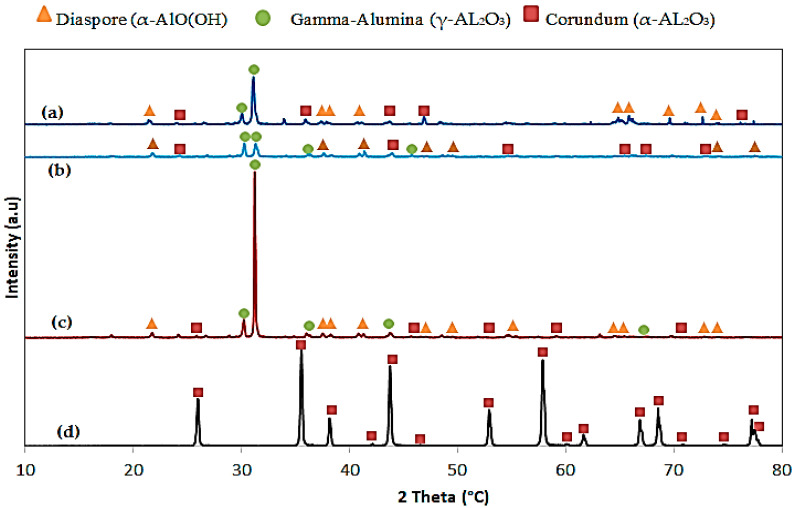
XRD pattern comparing the effect of reaction times on synthesis of Alumina; (**a**) AL-6-30, (**b**) AL-12-30, (**c**) AL-24-30 and (**d**) AL-Commercial.

The synthesized aluminum patterns of AL-6-30, AL-12-30, AL-24-30, and AL-Commercial are seen in Figure 2. Diaspore (α-AlO(OH)), boehmite (γ-AlO(OH)), aluminum oxides, or alumina (γ-Al_2_O_3_) crystalline structure and corundum have been found in all observed pinnacles of the synthesized alumina. In the wide-angle field, XRD patterns of mesoporous alumina indicated that the γ-Al_2_O_3_ process is 2*θ* (30.2°), 31.2° with wide peaks, 43.0°, and 67.0° (JCPDS card10-0425). There is no XRD peak in XRD patterns with AL-6-30, AL-12-30 and AL-24-30 synthesized alumina at a small angle of 1–10° which suggests that this mesoporous alumina is not structured by long-range pores. This suggests that there are no longer any long-range pores in this mesoporous alumina [26].

Diaspore, corundum, and gamma-alumina in the synthesis of alumina were described from the figure above XRD. Boehmite is a mineral of aluminum oxide (γ-AlO (OH)), which is an aluminum ore of bauxite [27]. According to Al’myasheva et al. [27], boehmite is also considered as a metastable phase throughout alumina transformation phases. It is dimorphous with diaspore. Diaspore is an aluminum oxide hydroxide (α-AlO(OH)) mineral, crystallizing in the orthorhombic system and isomorphous with goethite [28]. The structural collapse of diaspore occurs after hydrogen transfers and water extraction to be immediately transferred into gamma-alumina, which is considered the fast-track way to transform the alpha-alumina throughput diaspore phase [29]. Krokidis et.al. [29], also reported that after hydrogen transfers and water extraction, γ-alumina characteristics, the structural failure of boehmite is caused by an aluminum process of migration. The increase in the calcination temperature from the aluminum hydroxide gel started from gibbsite (γ-Al(OH)_3_) at temperature 100 °C then transferred into phase monohydrate to boehmite (γ-AlO(OH)) at around 150 °C. After that, the boehmite phase is a metastable phase that transformed into the diaspore (α-AlO(OH)) equilibrium phase at about 275 °C. Then corundum (Al_2_O_3_) appeared from the diaspore when the temperature reached 400 °C. Corundum is a crystalline form of aluminum oxide, typically containing traces of iron, titanium, vanadium, and chromium. Eventually, the transformation phases of alumina were completed to form the gamma-alumina phase (γ-AL_2_O_3_) which formed as the desired transformation alumina phase at the calcination of the corundum at 500 °C [30].

A lattice’s translational symmetry was combined with other symmetry elements, such as rotational and/or screw axes, to form the space-group symmetry [31]. The X-ray diffraction patterns were used to estimate the cellular structure, lattice parameters, and the position of Al and O atoms in α-AlO(OH), boehmite, aluminum oxides, and alumina [31]. The lattice types, usually in capital letters, are paired with the point group’s identification to produce a symbol for naming space groups [32,33]. Screw axes and glide planes in the lattice structure are also indicated, providing a full crystallographic space group. The aluminum oxides crystal structure exhibits hexagonal symmetry and the space group R3c (No.167). The structure is made up of a hexagonal tightly packed network of oxygen atoms along the [001] direction, having Al atoms taking up 2/3 (i.e., 67%) of the octahedral intercellular spaces [32]. Furthermore, alumina (γ-AlO(OH)) produced from boehmite has been reported to exhibit a cubic spinel structure with Fd 3 m space group symmetry, which corresponds to number 227 [33].

For alumina, AL-6-30 and-12-30 at room temperature and at lower aging times as shown in Figure 2, the structure with wider peaks seemed to be less crystalline, and alumina in the rooms at the higher aging time (AL-24-30, in the structure with widened peaks) seemed more crystalline. By contrast with AL-12-30, the alumina AL-6-30 revealed that the AL-6-30 showed a higher strength in the observable peaks. Although the pressure found of XRD result peaks relative to other synthesized alumina, AL-6-30 and AL-12-30 revealed the highest in the synthesis at room temperature and aging time and also that AL-24-30 was to be the strongest crystalline structure. The observable peaks were determined to be aluminum oxide or crystalline structure of alumina (Al_2_O_3_) for AL-commercial. There were many instances where the obvious peaks of Al_2_O_3_ were at 26.011°, 35.58°, 43.37°, 52.93° and 66.86°. Grain boundaries are crystal structural flaws that tend to lower the thermal and electrical conductivity of the alumina. Increases in grain boundaries have a movement phase of more than 11 °C. Furthermore, reduced grain boundaries have a movement phase of less than 11 °C. Thus, the synthesized alumina at room temperature and 24 h reaction time, AL-24-30, had the highest intensity for the observed peaks compared with the other two alumina. This means that alumina AL-24-30 had more crystalline structure due to the increase in reaction time with significant impact on the crystal structures and properties of synthesized alumina. Moreover, particles of aluminum hydroxide are solubilized, and growth occurs through condensation reaction during the aging process. Therefore, the alumina that aged for longer, AL-24-30, is granular in shape and well dispersed without obvious agglomerations that affect the formation of the synthesizing alumina, and this summary is quite compatible with FTIR results.

### 3.3. Morphological Characteristics of the Synthesized Alumina

**Figure 3:**
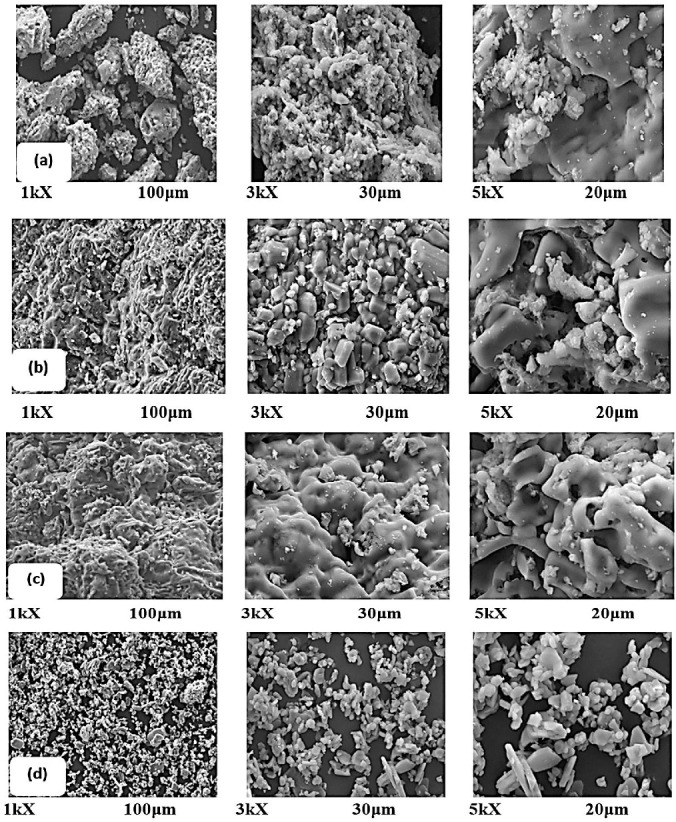
SEM micrographs comparing the effect of reaction times on synthesis of Alumina; (**a**) AL-6-30, (**b**) AL-12-30, (**c**) AL-24-30 and (**d**) AL-commercial.

Figure 3 displays the SEM micrographs of the synthesized alumina AL-6-30, AL-12-30, AL-24-30, and AL-commercial. The images below demonstrate that the solid morphologies of all synthesized samples were similar, with heterogeneous particle sizes.

All synthesized alumina had a non-spherical shape and appeared to have hexagonal-like shape particles. Identical results [34] were published. In the calcination, relatively small crystallites were bound to certain particles with a disordered structure [35]. In contrast to the superior morphology of AL-commercial alumina, synthesized alumina AL-6-30, AL-12-30, and AL-24-30. AL-commercial showed a smooth surface and spherical shapes with a larger grain size compared with the synthesized alumina. This may be due to different calcination conditions where higher calcination temperatures or longer calcination times were used in producing the commercial alumina. The morphological features revealed that the grain size increased by increasing the calcination temperature [34].

AL-24-30 had a longer aging time that led to an acceleration of the formation of larger structures. The SEM micrograph results of the synthesized alumina, which were obtained from the sol–gel method which was revealed to the morphology of the γ-Al_2_O_3_ nano-structured particles and a non-spherical shape [34]. The roughness of the surface can be caused by impurities [10]. It is worth remembering that the size of the particle distribution was not consistent, but we have shown that the particle sizes of the nano-metric and the micro-metric scales were different. The reactivity of the particles in a smaller size was more important on the nanometric scale [36].

## 4. Conclusions

In this study, the effect of reaction time on the synthesis of γ-alumina (from aluminum waste-cans) and their physicochemical characterizations was succinctly investigated. From the FTIR analysis, the formation of sharp peaks indicated better functional group characteristics of the aluminum and oxygen phase. Overall, the synthesized γ-alumina clearly revealed that the greater intensity of aluminum hydroxide and aluminum oxide indicates the presence of a better bond of alumina and oxygen to form aluminum oxide. Nevertheless, both XRD and FTIR results corroborated the higher intensity and more crystalline structure of the synthesized alumina. The SEM micrograph results of the synthesized alumina from the sol–gel method all shared almost similar heterogeneous particle size and non-spherical-shaped features. In a nutshell, the synthesized alumina at one-day aging time and room temperature attained the better optimum synthesis conditions compared with the commercial alumina. Consequently, research findings have demonstrated the performance of γ-alumina synthesis from aluminum waste containers.

## Data Availability

All the data are available within this manuscript.

## References

[1] Michel Devadoss P.S., Agamuthu P., Mehran S.B., Santha C., Fauziah S.H. (2021). Implications of Municipal Solid Waste Management on Greenhouse Gas Emissions in Malaysia and the Way Forward. Waste Manag..

[2] Zainu Z.A., Songip A.R. (2017). Policies, challenges and strategies for municipal waste management in malaysia. J. Sci. Technol. Innov. Policy.

[3] Goh Y.G., Sufian A.S., Mohamad N., Afiqah N. (2020). Mechanical Properties of Recycled Polypropylene Filled with Aluminium Hydroxide. Mater. Sci. Forum.

[4] Umar U.A., Shafiq N., Ahmad F.A. (2021). A Case Study on the Effective Implementation of the Reuse and Recycling of Construction & Demolition Waste Management Practices in Malaysia. Ain Shams Eng. J..

[5] Baarimah A.O., Syed Mohsin S.M., Alaloul W.S., Ba-naimoon M.S. (2021). Effect of Sodium Hydroxide on Mechanical Characteristics of Kenaf Fibers Reinforced Concrete. J. Phys. Conf. Ser..

[6] Syed Mohsin S.M., Baarimah A.O., Jokhio G.A. (2018). Effect of Kenaf Fiber in Reinforced Concrete Slab. IOP Conf. Ser. Mater. Sci. Eng..

[7] Brough D., Jouhara H. (2020). The Aluminium Industry: A Review on State-of-the-Art Technologies, Environmental Impacts and Possibilities for Waste Heat Recovery. Int. J. Thermofluids.

[8] Bashir M.J.K., Jun Y.Z., Yi L.J., Abushammala M.F.M., Amr S.S.A., Pratt L.M. (2020). Appraisal of Student’s Awareness and Practices on Waste Management and Recycling in the Malaysian University’s Student Hostel Area. J. Mater. Cycles Waste Manag..

[9] Bin Mokaizh A.A., Wirman N., Shariffuddin J.H. (2019). Synthesis of Alumina from Aluminium Can Waste to Be Applied as Catalyst Support for Biodiesel Production. IOP Conf. Ser. Mater. Sci. Eng..

[10] Sheel T.K., Poddar P., Murad A., Neger A., Chowdhury A.M.S. (2016). Preparation of Aluminum Oxide from Industrial Waste Can Available in Bangladesh Environment: SEM and EDX Analysis. J. Adv. Chem. Eng..

[11] Adans Y.F., Martins A.R., Coelho R.E., das Virgens C.F., Ballarini A.D., Carvalho L.S. (2016). A Simple Way to Produce γ-Alumina From Aluminum Cans by Precipitation Reactions. Mater. Res..

[12] Ahmedzeki N.S., Hussein S., Abdulnabi W.A. (2017). Recycling Waste Cans to Nano Gamma Alumina: Effect of the Calcination Temperature and PH. Int. J. Curr. Eng. Technol.

[13] Hamid S., Mat Isa C.M., Felix S.N., Mustaffa N.K. (2020). Sustainable Management Using Recycle and Reuse of Construction Waste Materials in Malaysia. ESTEEM Acad. J..

[14] Matori K., Wah L., Hashim M., Ismail I., Zaid M. (2012). Phase Transformations of α-Alumina Made from Waste Aluminum via a Precipitation Technique. Int. J. Mol. Sci..

[15] Bin Mokaizh A.A., Shariffuddin J.H.B.H. (2021). Synthesis of α-Alumina Developed from Waste Aluminium Using Precipitation Technique. J. Phys. Conf. Ser..

[16] Abdellah W., Abdelfattah E., Diab H., Saad E. (2018). Removal of Chromium from Liquid Waste by Gamma Aluminum Oxide (γ-Al_2_O_3_) Nanoparticles Synthesized Using Citrate Sol–Gel Method. Arab J. Nucl. Sci. Appl..

[17] Omer A.H., Bin Mokaizh A.A., Shariffuddin J.H.B.H. (2021). Low-Calcination Temperature to Synthesize A-Alumina From Aluminium Waste Can Using Sol-Gel Method. IOP Conf. Ser. Earth Environ. Sci..

[18] Bin Mokaizh A.A., Al Haiqi O., Binti Haji Shariffuddin J.H. (2021). Investigating the Effects of Calcination Time on A-Alumina Synthesis from Aluminum Waste Can. Phys. Chem. Earth Parts A/B/C.

[19] Bin Mokaizh A.A., Shariffuddin J.H.B.H., Makhlouf A.S.H., Ali G.A.M. (2021). Manufacturing of Nanoalumina by Recycling of Aluminium Cans Waste.

[20] Liu R., Xu T., Wang C. (2016). A Review of Fabrication Strategies and Applications of Porous Ceramics Prepared by Freeze-Casting Method. Ceram. Int..

[21] Hosseini S.Y., Khosravi-Nikou M.R. (2016). Synthesis and Characterization of Nano-Sized γ-Al 2 O 3 for Investigation the Effect of Temperature on Catalytic Dehydration of Methanol to Dimethyl Ether. Energy Sources Part A Recover. Util. Environ. Eff..

[22] Rajaeiyan A., Bagheri-Mohagheghi M.M. (2013). Comparison of Sol-Gel and Co-Precipitation Methods on the Structural Properties and Phase Transformation of γ and α-Al2O3 Nanoparticles. Adv. Manuf..

[23] Segal F.M., Correa M.F., Bacani R., Castanheira B., Politi M.J., Brochsztain S., Triboni E.R. (2018). A Novel Synthesis Route of Mesoporous γ-Alumina from Polyoxohydroxide Aluminum. Mater. Res..

[24] Bazyari A., Mortazavi Y., Khodadadi A.A., Thompson L.T., Tafreshi R., Zaker A., Ajenifujah O.T. (2016). Effects of Alumina Phases as Nickel Supports on Deep Reactive Adsorption of (4,6-Dimethyl) Dibenzothiophene: Comparison between γ, δ, and θ-Alumina. Appl. Catal. B Environ..

[25] Xu N., Liu Z., Bian S., Dong Y., Li W. (2016). Template-Free Synthesis of Mesoporous γ-Alumina with Tunable Structural Properties. Ceram. Int..

[26] Chotisuwan S., Sirirak A., Har-Wae P., Wittayakun J. (2012). Mesoporous Alumina Prepared from Waste Aluminum Cans and Used as Catalytic Support for Toluene Oxidation. Mater. Lett..

[27] Al’myasheva O.V., Korytkova E.N., Maslov A.V., Gusarov V. (2005). V Preparation of Nanocrystalline Alumina under Hydrothermal Conditions. Inorg. Mater..

[28] Kononchuk O., Alekseev A., Zubkova O., Udovitsky V. (2017). Scientific Background for Processing of Aluminum Waste. E3S Web Conf..

[29] Krokidis X., Raybaud P., Gobichon A.-E., Rebours B., Euzen P., Toulhoat H. (2001). Theoretical Study of the Dehydration Process of Boehmite to γ-Alumina. J. Phys. Chem. B.

[30] Lamouri S., Hamidouche M., Bouaouadja N., Belhouchet H., Garnier V., Fantozzi G., Trelkat J.F. (2017). Control of the γ-Alumina to α-Alumina Phase Transformation for an Optimized Alumina Densification. Boletín La Soc. Española Cerámica Vidr..

[31] Noel Y., Demichelis R., Pascale F., Ugliengo P., Orlando R., Dovesi R. (2009). Ab Initio Quantum Mechanical Study of γ-AlOOH Boehmite: Structure and Vibrational Spectrum. Phys. Chem. Miner..

[32] Ramogayana B., Santos-Carballal D., Maenetja K.P., de Leeuw N.H., Ngoepe P.E. (2021). Density Functional Theory Study of Ethylene Carbonate Adsorption on the (0001) Surface of Aluminum Oxide α-Al_2_O_3_. ACS Omega.

[33] Bradley C., Cracknell A. (2010). The Mathematical Theory of Symmetry in Solids: Representation Theory for Point Groups and Space Groups.

[34] Asencios Y.J.O., Sun-Kou M.R. (2012). Synthesis of High-Surface-Area γ-Al2O3 from Aluminum Scrap and Its Use for the Adsorption of Metals: Pb(II), Cd(II) and Zn(II). Appl. Surf. Sci..

[35] Ezzuldin S.S.M., Rahim S.B.A., Wan Yussof H., Olalere O.A., Habeeb O.A. (2019). Morphological, Thermal Stability and Textural Elucidation of Raw and Activated Palm Kernel Shell and Their Potential Use as Environmental-Friendly Adsorbent. Chem. Data Collect..

[36] Vargas-Martínez N., Ruíz-Baltazar Á.d., Medellín-Castillo N.A., Reyes-López S.Y. (2018). Synthesis of α-Alumina Nano-Onions by Thermal Decomposition of Aluminum Formate. J. Nanomater..

